# Host Defense and Tolerance: Unique Challenges in the Placenta

**DOI:** 10.1371/journal.ppat.1002804

**Published:** 2012-08-09

**Authors:** Varvara B. Zeldovich, Anna I. Bakardjiev

**Affiliations:** 1 Department of Pediatrics, University of California, San Francisco, San Francisco, California, United States of America; 2 Program in Microbial Pathogenesis and Host Defense, University of California, San Francisco, San Francisco, California, United States of America; Duke University Medical Center, United States of America

## What Are the Unique Challenges of Mammalian Gestation?

Mammalian gestation creates an immunological paradox wherein the body must balance tolerance of an allogeneic fetus with protection against invading pathogens. Pregnancy has long been considered a state of immune suppression that, while necessary for reproduction, increases a woman's susceptibility to infection [1]. However, mothers bear the responsibility of the most important biological task: to carry and to nourish their offspring. It is therefore fitting that a more nuanced picture is emerging of a tightly regulated maternal immune system that balances awareness of the fetus with mechanisms to protect against pathogens and to sustain a healthy pregnancy [2]. Recent evidence indicates that innate placental defenses comprise one such mechanism. Subversion of these defenses by pathogens can lead to pregnancy complications such as preterm labor or vertical transmission with fetal morbidity or mortality [3]. The Danger Model posits that tissue damage rather than foreign antigens trigger inflammation [4]. This notion encompasses the paradox of mammalian pregnancy and suggests that infection-based loss of placental integrity may be the route to complications. Here we review recent evidence for innate placental barriers to infection and how these can be breached by pathogens.

## What Is the Function and Structure of the Placenta?

**Figure 1:**
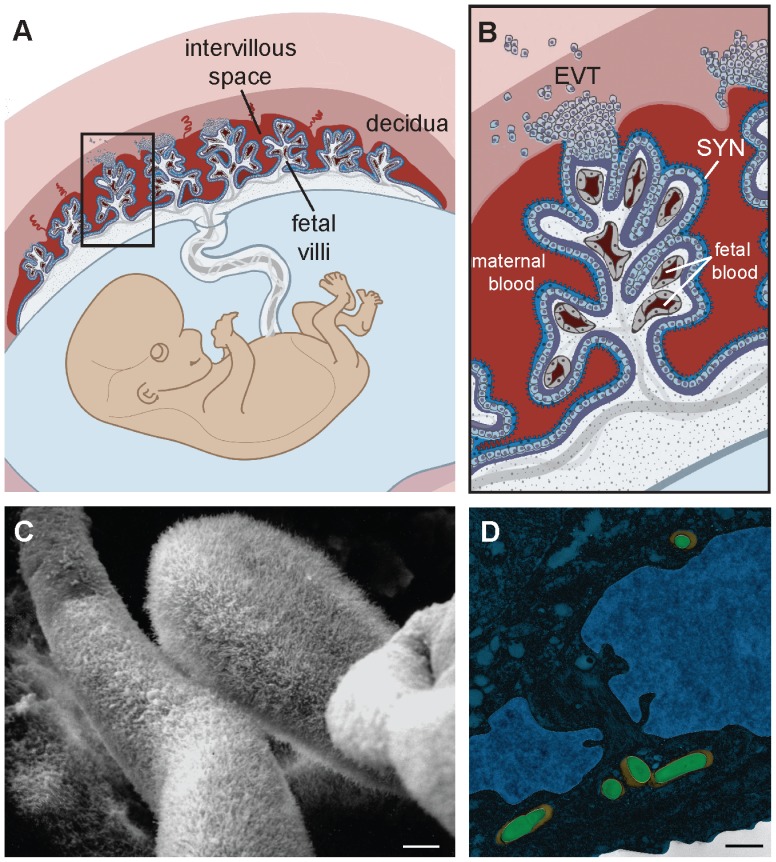
Structure and barriers of the human maternal-fetal interface. (A) Structure of the human placenta. Maternal and fetal tissues are in tones of red and blue, respectively. Adapted from [6]. See text for detailed explanation. (B) Enlargement of boxed area in panel A showing points of direct contact between mother and fetus. EVT, extravillous trophoblasts; SYN, syncytium. Adapted from [6]. (C) Scanning electron micrograph of first trimester human placenta showing the syncytial surface with profuse branched microvilli (courtesy of Susan Fisher, UCSF). (D) False-colored transmission electron micrograph of primary human EVT. *Listeria monocytogenes* (green) is trapped in endolysosomes (orange). Adapted from [23]. Bar, 1 µm.

The placenta is a chimeric organ made of maternal and fetal cells and has two main functions: to nourish and to protect the fetus. Understanding the structure of the placenta is key to understanding its functions (Figure 1A–B). Specialized fetally derived cells called trophoblasts differentiate into several subpopulations that perform critical placental functions. In humans, invasive extravillous trophoblasts (EVT) anchor the placenta in the uterine implantation site (decidua) where they are juxtaposed to maternal immune cells. EVT also invade and restructure maternal arteries to facilitate blood flow to the fetus; specifically, maternal blood flows into the intervillous space, where it bathes fetally derived villous trees. The surface of these villi consists of a syncytium, a fused multinucleate trophoblast layer that mediates nutrient and gas exchange between maternal and fetal tissues. To facilitate such transport its surface is covered by branched microvilli (Figure 1C), whose total surface area reaches approximately 12 m^2^ by the end of human gestation. While facilitating transport from mother to fetus, the syncytium must also protect the offspring from blood-borne pathogens. Indeed, the syncytium is resistant to infection with multiple diverse microbes that are important human pathogens during pregnancy (e.g., cytomegalovirus [CMV] [5], the bacterium *Listeria monocytogenes* [6], and the protist *Toxoplasma gondii* [7]).

It is important to note that placental structure differs between mammalian species (reviewed in [8]), which influences the relevance of in vivo models for human disease. For example, there are a number of differences between the human and mouse placenta that limit the utility of the mouse as an experimental model. Nevertheless, pregnant mice are used because of the availability of tools to study the host immune response to infection. In addition, isolated murine placental cell types that recapitulate human phenotypes might prove useful in the study of host-pathogen interactions in the placenta.

## How Does the Syncytium Protect the Fetus from Blood-Borne Pathogens?

In order to cause infection pathogens have to be able to (a) adhere, (b) invade, (c) replicate, and/or (d) avoid elimination. The barrier created by the syncytium against diverse pathogens suggests that its general cell biological properties may interfere with these steps of microbial colonization and growth.

In order to understand how the syncytium resists microbial colonization, it is useful to compare the syncytium to other epithelial surfaces that can be breached by pathogens. For example, gastrointestinal pathogens typically use receptors that are components of intercellular junctions to breach the polarized epithelium of the intestinal barrier [9]. The formation of a fused multinucleate syncytium that foregoes intercellular junctions may therefore have evolved as a defense mechanism. This is illustrated by studies of the food-borne pathogen *L. monocytogenes*, which breaches the intestinal barrier via the interaction of the virulence determinant internalin A with the host cell receptor E-cadherin that is transiently exposed at the tips of intestinal villi [10], [11]. E-cadherin is typically not expressed on the apical surface of any epithelia, and thus its paucity of expression on the blood-bathed surface of the syncytium [6], [12] coupled with the lack of intercellular junctions makes adherence and internalization of *L. monocytogenes* via this route unlikely. Entry of herpes simplex virus into the syncytium is similarly inefficient, largely due to the lack of its host cell receptors HveA, HveB, and HveC [13]. Of note, the lack of intercellular junctions may also prevent transmigration of maternal blood leukocytes into the fetus [14], a process that must be tightly controlled for the sake of tolerance. In addition, the syncytium has been shown to be resistant to infection by cell-to-cell spread of *L. monocytogenes* from infected macrophages [6], and direct invasion by *T. gondii* [7]. Surface receptors have not yet been identified for these processes, and it is possible that additional mechanisms contribute to the resistance of this specialized epithelium. For example, the network of profuse branched microvilli (Figure 1C) might preclude adherence of microbes as well as infected leukocytes.

Other explanations must be sought for the apparent resistance of the syncytium to infection from the basal side. *L. monocytogenes*, *T. gondii*, and CMV can all be found in cytotrophoblasts underlying uninfected syncytium [5]–[7]. The basal surface of the syncytium may be guarded against invasion by the dense cytoskeletal network that supports such a laterally vast multinucleate cell [9]. The apical to basal directionality of nutrient transport machineries [15] may also preclude endocytic uptake of pathogens on the basal side. Furthermore, host cell invasion efficiency by *L. monocytogenes* has recently been linked to abundance of fused mitochondria [16]. Thus, the unusually fragmented state of mitochondria in the syncytium [17] may explain low invasion rates. Finally, syncytial production of reactive nitrogen species has been hypothesized to reduce invasion and intracellular growth of *Trypanosoma cruzi* [18] and may contribute to elimination of other pathogens as well.

Thus, the syncytium creates a formidable barrier to infection by virtue of multiple unique cell biological properties. Furthermore, its function as a protective layer against blood-borne microbes and transmigration of maternal leukocytes may be the reason why all hemochorial placentas have evolved a syncytium [8].

## What Is the Role of Extravillous Trophoblasts in Placental Defenses?

T he resistance of the syncytium to infection begs the question where microbes breach the maternal-fetal barrier. The other point of direct contact between maternal and fetal cells is formed by EVT invading deep into the uterine implantation site, which contains an abundance of maternal leukocytes [2]. Macrophage precursors from maternal blood are actively recruited to the implantation site in the pregnant mouse model [19]. Is it possible for pathogens to hitch a ride? Indeed, multiple observations point in this direction. First, all pathogens that are known to infect the placenta and/or fetus have intracellular life cycles (Table 1) [20], and most are able to infect and survive in leukocytes. Second, multiple studies demonstrate that intrauterine infection in vivo with a variety of different pathogens, including *L. monocytogenes* [21] and *T. gondii* [22], begins in the implantation site. Third, we have recently shown that EVT are the preferred site of entry for *L. monocytogenes* and *T. gondii* in primary human placental organ cultures [6], [7].

**Table 1:** Placental pathogens.

Bacteria	Parasites	Viruses
*Brucella* spp.	*Plasmodium falciparum*	Cytomegalovirus
*Coxiella burnetii*	*Toxoplasma gondii*	Rubella virus
*Listeria monocytogenes*	*Trypanosoma cruzii*	Parvovirus B19
*Mycobacterium tuberculosis*	*Leishmania* spp.	Varicella zoster virus
*Salmonella typhi*		Lymphocytic choriomeningitis virus
*Treponema pallidum* a		

a Generally thought to be extracellular, but has been documented in intracellular compartments. For a more detailed review of placental pathogens, their route of horizontal transmission, and host range, please see [20].

However, EVT also have strong innate defense mechanisms against intracellular pathogens. We observed that spread of *L. moncytogenes* beyond EVT into deeper layers of primary human placental organ cultures was hindered [6]. In addition, isolated primary human EVT entrap *L. monocytogenes* in lysosomal compartments where they are degraded (Figure 1D) [23]. EVT appear to be restrictive for viral growth as well. Recent studies indicate that the majority of HIV-1 virions are trapped within endosomal compartments in trophoblasts, and that EVT inactivate HIV replication mechanisms [24], [25].

These experimental systems suggest that innate host defense mechanisms in EVT may hinder the normal life cycle of intracellular pathogens and prevent microbial growth and spread. It is possible that the invasive role of EVT and their active breakdown of extracellular matrix may require unique degradative and endolysosomal pathways that interfere with the life cycle of intracellular pathogens. Moreover, placental production of antimicrobials like β-defensins, indoleamine 2,3-dioxygenase, cathelicidin, and reactive oxygen and nitrogen species has been established [26] and may be responsible for EVT resistance to pathogens.

## How Can Infection Progress and Lead to Pregnancy Complications?

Despite the effectiveness of the placental barrier, infections can nevertheless progress to cause pregnancy complications such as spontaneous abortion and preterm labor. While the molecular mechanisms of preterm labor are still poorly understood, it is associated with placental inflammation that may be triggered by infection and/or loss of placental integrity [3]. What factors contribute to the occasional progression of placental and fetal infection?

The “Danger model” suggests that the maternal immune system reacts to the presence of danger signals [4] and provides a plausible explanation for the paradox of mammalian gestation. The syncytial and EVT barriers may be effective until a certain threshold of cellular damage is accrued. One possibility to reach this threshold occurs when several insults such as co-infection with multiple pathogens challenge the defense mechanisms of the placenta. Consistent with this model are the findings that human preterm placentas are often colonized with multiple microbes [27]. Similarly, histopathological analyses reveal that CMV is more often found in human placentas with concurrent bacterial infections [28], and more recently it has been shown that viral infection and bacterial products synergize to trigger preterm labor in the pregnant mouse model [29]. Another example is placental infection with *Plasmodium falciparum*, the causative agent of malaria. Parasite-infected erythrocytes accumulate in the intervillous space [30], which may lead to damage of the syncytium [31], and increased rates of vertical transmission of HIV-1 [32].

In summary, the placenta has developed powerful defenses against infection consisting of multiple layers of unique cell biological barriers. These innate safeguards dovetail with the modulated immune system during pregnancy to balance the need for tolerance with protection against pathogens. Damage of the feto-placental unit beyond a certain threshold triggers the termination of pregnancy—a sensible defense for mother and species.
